# Shortlisting SARS‐CoV‐2 Peptides for Targeted Studies from Experimental Data‐Dependent Acquisition Tandem Mass Spectrometry Data

**DOI:** 10.1002/pmic.202000107

**Published:** 2020-06-21

**Authors:** Duarte Gouveia, Lucia Grenga, Jean‐Charles Gaillard, Fabrice Gallais, Laurent Bellanger, Olivier Pible, Jean Armengaud

**Affiliations:** ^1^ Université Paris Saclay, CEA, INRAE Département Médicaments et Technologies pour la Santé (DMTS), SPI Bagnols‐sur‐Cèze 30200 France

**Keywords:** COVID‐19, mass spectrometry, peptides, proteomics, SARS‐CoV‐2, viral protein detection

## Abstract

Detection of severe acute respiratory syndrome coronavirus 2 (SARS‐CoV‐2) is a crucial tool for fighting the COVID‐19 pandemic. This dataset brief presents the exploration of a shotgun proteomics dataset acquired on SARS‐CoV‐2 infected Vero cells. Proteins from inactivated virus samples were extracted, digested with trypsin, and the resulting peptides were identified by data‐dependent acquisition tandem mass spectrometry. The 101 peptides reporting for six viral proteins were specifically analyzed in terms of their analytical characteristics, species specificity and conservation, and their proneness to structural modifications. Based on these results, a shortlist of 14 peptides from the N, S, and M main structural proteins that could be used for targeted mass‐spectrometry method development and diagnostic of the new SARS‐CoV‐2 is proposed and the best candidates are commented.

The world is facing a tremendous pandemic caused by the SARS‐CoV‐2 virus.^[^ ^1^ ^]^ The mutations naturally‐occurring in the viral genome upon its spread may challenge molecular biology diagnostic tests.^[^ ^2^, ^3^ ^]^ Mass‐spectrometry (MS) based detection of organisms could be an alternative for fast deployment of novel detection means in case of emergence of new pathogens. More specifically, proteotyping based on peptide/protein mass measurement can be performed with either a targeted strategy or without any a priori.^[^ ^4^, ^5^ ^]^ For such purpose sample preparation, acquisition modes and parameters should be optimized depending on the nature of the samples and the target. Targeted mass spectrometry approaches can be applied for the routine detection of pathogens with high sensitivity.^[^ ^6^ ^]^ Previous knowledge of peptide sequences and experimental parameters is required for the development of robust methods.

This article reports high‐resolution mass spectrometry (HR‐MS) experimental data from SARS‐CoV‐2 infected Vero cells and proposes a list of experimentally observed peptides for their possible use in targeted method development. We recently described the kinetic of Italy‐INMI1 SARS‐CoV‐2 virus production in Vero E6 cells and pointed at the key host proteins whose abundances are correlated with those of viral proteins.^[^ ^7^ ^]^ One sample of this kinetic, at 4 days post‐infection (4dpi) at Multiplicity of infection of 0.01, was subjected to a deeper data‐dependent acquisition (DDA) analysis. The spectra obtained were explored to identify and retrieve important experimental characteristics of peptides belonging to the most abundant viral proteins. The 4dpi sample was selected based on our previous results that show a high load of viral protein production at this stage in these conditions. Briefly, SARS‐CoV‐2‐infected Vero E6 cells cultivated in a BSL3 facility in DMEM supplemented with 5% foetal calf serum and 0.5% penicillin‐streptomycin at 37 °C under 9% CO_2_ were washed and inactivated at 125 °C for 40 min. Proteins were dissolved in 100 µL LDS 1X (Lithium dodecyl sulfate) sample buffer (Invitrogen) and supplemented with 5% beta‐mercaptoethanol (v/v). For the shotgun analysis, two 20 µL aliquots of extracted proteins were loaded onto a NuPAGE 4–12% bis–tris gel (technical replicates) and subjected to electrophoretic migration at 200 V for 10 min. The proteins were stained for 5 min with Coomassie SimplyBlue SafeStain (Thermo Fisher Scientific). Each gel band was then sliced along their molecular weights in five fractions which were subjected to trypsin proteolysis^[^ ^8^ ^]^ and analyzed separately by liquid chromatography (LC) HR‐MS/MS with a Q‐Exactive HF mass spectrometer. The 90 min LC gradient comprised two slopes of solvent B (80% acetonitrile, 0.1% formic acid in water): 4–25% during 75 min, and 25–40% during 15 min. The MS/MS spectra were assigned to peptide sequences using the Mascot Server 2.5.1 (Matrix Science) against an “in‐house” contaminants database (cRAP + additional contaminants + 23 *Bos Taurus* sequences; 384 sequences; 187 250 residues), followed by a search against a database comprising the Uniprot *Chlorocebus* sequences (20 576 sequences; downloaded March 2020) and the Italy‐INMI1 SARS‐CoV‐2 protein sequences (10 sequences; downloaded March 2020). The following parameters were used for peptide assignation: trypsin as enzyme, two missed cleavages allowed, precursor charges of +2 and +3, mass tolerances of 5 ppm for the precursor ions and 0.02 Da for the fragment ions, carbamidomethylated cysteines as static modification, and methionine oxidation, asparagine, and glutamine deamidation as dynamic modifications. Mascot DAT files were processed for filtering peptide‐to‐spectrum matches (PSMs) with false discovery rates inferior to 1%. Proteins identified by one or more specific peptides were retained and a label‐free quantification based on PSM counts for each peptide and protein was performed following the principle of parsimony. The mass spectrometry proteomics data have been deposited to the ProteomeXchange Consortium via the PRIDE^[^ ^9^ ^]^ partner repository with the dataset identifier PXD018804 and 10.6019/PXD018804.

Table S1, Supporting Information contains the list of proteins and peptides identified. The ten nanoLC‐MS/MS runs originated a total of 485 396 MS/MS spectra, from which 167 048 were attributed to 16 929 non‐redundant peptide sequences. This data allowed identifying and quantifying 2900 protein sequences from the whole‐cell content of the sample. The viral proteome represents a small but valuable fraction of the dataset: 3363 PSMs (2%), 101 peptide sequences (0.6%), and six proteins (0.2%). The six viral proteins identified in these samples were proteins N (Nucleoprotein—39 peptides), S (Spike protein—31 peptides), M (membrane glycoprotein—eight peptides), ORF1ab (12 peptides), ORF3a (three peptides), and ORF8 (one peptide). To propose the most suitable viral peptides for the development of targeted approaches, we analyzed the dataset within the Skyline software (v20.1.0.76, University of Washington).^[^ ^10^ ^]^ We created a spectral library based on the two DAT files from the Mascot search (cut‐of 0.95) and uploaded the MS1 full scan information contained in the ten RAW files. The protein database previously used for the Mascot search was used as background proteome. Only the viral proteins were added to the target panel. The peptide settings were then changed as follows: trypsin as enzyme; no missed cleavages; enforce peptide uniqueness by species; use measured retention times; peptide length from seven to 25 amino acids; no structural modifications; and pick peptides matching the library. For transitions: precursor charges of one and three; ion charges of one; ion types p, b, and y; up to five product ions picked; ion match tolerance = 0.05 *m*/*z*; method match tolerance = 0.055 *m*/*z*; MS1 filtering centroided with 5 ppm of mass accuracy; MS/MS filtering with a resolving power of 15 000 at 200 *m*/*z*. These parameters output a list of 300 transitions, 60 precursor ions, 53 peptides reporting for five viral proteins. Peak peaking was manually checked for all peptides. To remove peaks with unreliable isotope patterns, possible misidentifications, or signals below the limit of detection with excessive noise or interferences, we further optimized the peptide filtering by selecting only precursors with an isotope dot products (idotp) superior to 0.90. The idotp provides a measure to assess precursor isotope distribution and its correlation between the predicted and the observed pattern, with “1” being an optimal match.^[^ ^11^ ^]^

The final list of peptides, precursor, and fragment ions is presented in **Table** **1**. The table shows the sequences of the precursor ions, their charges and *m*/*z*, and the top three ranked transitions in the library. The corresponding Skyline LC peaks and MS/MS spectra for each of the 16 precursor ions from Table 1 are available in SD1, Supporting Information. As shown in Table 1, protein N is the most represented with seven peptides, followed by protein S with four peptides, and protein M with three peptides. The retention times and peptide intensities in the chromatogram are represented in **Figure** **1**. The figure shows that these 14 peptides are distributed essentially in three zones of the chromatogram: five peptides in the first 25 min; four peptides in the 38–50 min window; and five peptides in the 75–85 min window. The five early‐eluting peptides could be of great interest for proteotyping viral peptides by concentrating the mass spectrometry efforts on their detection. If their elution could be achieved earlier, extra‐short LC gradients could be developed for their targeted detection. Moreover, these five peptides report for the three major proteins of the virus (two peptides for proteins N and M, one peptide for protein S). In terms of abundance, all peptides were observed with high intensities at least in one of the fractions (peak areas between 10^7^ and 10^8^). The five precursor ions that showed highest peak intensities were: AYNVTQAFGR (+2), GFYAEGSR (+2), ADETQALPQR (+2) from protein N, and peptides EITVATSR (+2) and VAGDSGFAAYSR (+2) from protein M. For protein S, the most intense precursor was LQSLQTYVTQQLIR (+2), which correspond to the seventh most intense peak from the list. The less abundant precursors from this list were GWIFGTTLDSK (+2, protein S), IAGHHLGR (+2, protein M), WYFYYLGTGPEAGLPYGANK (+3, protein N). More detailed information on precursors and transitions can be found on the two reports extracted from skyline (Tables S2 and S3, Supporting Information).

**Table 1:** List of the 14 viral peptides shortlisted for targeted method and their analytical characteristics, specificity, modifications, and missed cleavages

Protein	Peptide Sequence	Precursor Charge	Retention time [min]	Precursor *m*/*z*	Product *m*/*z* from top three ranked transitions	Inter‐species specificity^a)^	Intra‐species conservation^b)^	Modifications observed in the DDA data	Modifications observed in other deposited datasets^c)^	Estimation of modification rate based on GPMDB data	Detection of missed cleavages in the DDA data
Protein M	EITVATSR	2	19.48 ± 0.55	438.742899	434.235772 (y4), 634.351865 (y6), 175.118952 (y1)	no	yes	no	No	0%	EITVATSRTLSYYK
	IAGHHLGR	2	7.22 ± 0.32	430.74611	482.283391 (y4), 345.22448 (y3), 676.363767 (y6)	no	yes	no	No	0%	no
	VAGDSGFAAYSR	2	35.43 ± 0.75	600.785827	1030.458849 (y10), 171.112804 (b2), 858.410442 (y8)	yes	yes	no	One succinylation in 128 observations	1%	LGASQRVAGDSGFAAYSR, VAGDSGFAAYSRYR
Protein N	ADETQALPQR	2	21.33 ± 0.65	564.785827	400.230293 (y3), 584.351471 (y5), 187.071333 (b2)	yes	no	deamidation (NQ)	Eight deamidation in 87 observations	9%	ADETQALPQRQK, ADETQALPQRQKK, KADETQALPQRQR, KADETQALPQRQRQK, KKADETQALPQR
	AYNVTQAFGR	2	38.7 ± 0.77	563.78563	679.352199 (y6), 892.463541 (y8), 349.150646 (b3)	yes	no	deamidation (NQ)	11 deamidations in 139 observations	8%	RTATKAYNVTQAFGR, TATKAYNVTQAFGR
	GFYAEGSR	2	20.91 ± 0.56	443.706317	519.252151 (y5), 682.315479 (y6), 448.215037 (y4)	no	yes	no	Two phosphorylation in 33 observations	6%	GFYAEGSRGGSQASSR
	GPEQTQGNFGDQELIR	3	49.45 ± 0.77	596.955223	830.436657 (y7), 175.118952 (y1), 658.38825 (y5)	no	no	deamidation (NQ)	Eight deamidations in 98 observations	8%	RGPEQTQGNFGDQELIR
	IGMEVTPSGTWLTYTGAIK	3	82.63 ± 0.2	675.683564	753.414131 (y7), 866.498195 (y8), 652.366452 (y6)	yes	no	Oxidation (M)	Six carbamidomethyl, three phosphorylation, 30 dioxidation, 224 oxidation in 400 observations	66%	no
	NPANNAAIVLQLPQGTTLPK	2	76.11 +/‐ 0.69	1030.578571	841.477794 (y8), 1294.772913 (y12), 1195.704499 (y11)	yes	no	deamidation (NQ)	95 deamidation, seven phophorylation in 415 observations	25%	DHIGTRNPANNAAIVLQLPQGTTLPK
		3	76.12 +/‐ 0.69	687.388139	841.477794 (y8), 766.384228 (b8), 865.452642 (b9)						
	WYFYYLGTGPEAGLPYGANK	3	85.3 +/‐ 0.17	756.365111	649.330401 (y6), 819.435929 (y8), 890.473043 (y9)	yes	no	deamidation (NQ)	Two deamidations, 75 dioxidations, 75 oxidations in 443 observations	34%	WYFYYLGTGPEAGLPYGANKDGIIWVATEGALNTPK
Protein S	FQTLLALHR	3	47.82 +/‐ 0.79	366.885464	496.299041 (y4), 609.383105 (y5), 425.261928 (y3)	yes	yes	no	no	0%	no
	GWIFGTTLDSK	2	75.84 +/‐ 0.64	612.816595	244.108053 (b2), 868.441074 (y8), 721.37266 (y7)	yes	no	no	15 dioxidation, 11 oxidation in 52 observations	50%	no
	HTPINLVR	2	22.94 +/‐ 0.55	475.282526	711.451185 (y6), 239.113866 (b2), 175.118952 (y1)	yes	yes	deamidation (NQ)	Two deamidations in 51 observations	4%	no
	LQSLQTYVTQQLIR	2	78.5 +/‐ 0.69	845.977961	1121.631334 (y9), 1449.806004 (y12), 1249.689912 (y10)	yes	yes	no	no	0%	no
		3	78.5 +/‐ 0.69	564.321066	758.451913 (y6), 857.520327 (y7), 242.149918 (b2)						

a Specificity is given by hits only on human‐virus, the SARS‐CoV2, in a NCBInr BLAST search at 100% ID and 100% query coverage (on 24th of April 2020);

b 100% sequence conservation in human‐viruses from Figure 2;

c from Global Proteome Machine Database (GPMDB, https://gpmdb.thegpm.org/thegpm‐cgi/dblist_pep.pl)

**Figure 1:**
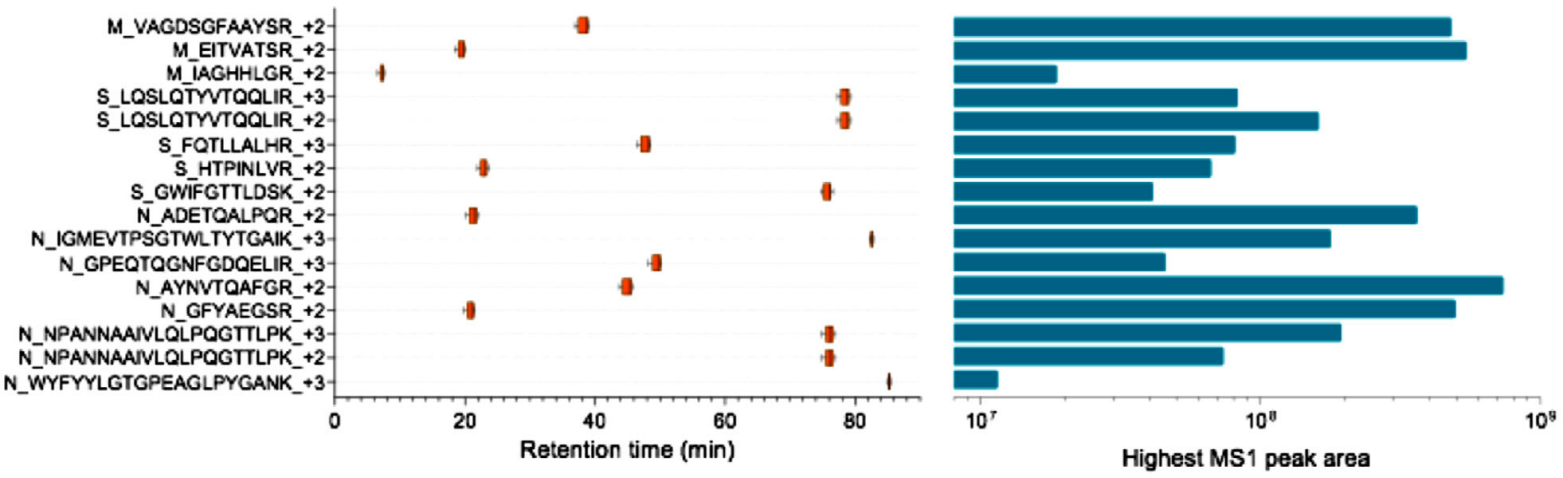
Retention times and peak areas from the 16 precursor ions identified from the skyline analysis.

Next, we verified peptide inter‐species specificity and sequence conservation among proteins N, M, and S from different human and animal SARS‐CoV‐2. As shown in Table 1, blastp^[^ ^12^ ^]^ results at 100% query coverage and identity show that ten peptides are specific to SARS‐CoV‐2, if one does not take into account the hits on bat and pangolin coronaviruses (see column “Inter‐species specificity”). However, peptides EITVATSR, IAGHHLGR, GFYAEGSR, and GPEQTQGNFGDQELIR are shared with other organisms and should be avoided for detection purposes. For assessing the intra‐species conservation of peptide sequences, 3,217 genomes were downloaded from the GISAID website (https://www.gisaid.org/) on the 2nd of April 2020. After multiple sequence alignment and curation against a reference genome assembly (GCA_009858895.3), genes were annotated using the corresponding GFF file (GCA_009858895.3_ASM985889v3_genomic.gff) and translated into proteins. Proteomes with more than two undetermined residues “X” due to nucleotide uncertainty in one of the 10 target protein sequences were eliminated, and 2005 curated proteomes were kept in the alignment. All the peptide sequence variants found in this analysis for the most mass spectrometry detectable candidates are displayed in **Figure** **2**. The three peptides from protein M are well‐conserved and no variants were found. Among the four peptides identified for protein S, peptide GWIFGTTLDSK shows some slight sequence variability in one human‐virus sequence (USA|WA‐UW370), and four non‐human‐virus sequences (pangolin sequences). Peptides HTPINLVR, LQSLQTYVTQQLIR and FQTLLALHR are well‐conserved among human‐virus and no variants were found. The peptides from protein N presented the highest diversity in terms of sequence. While the majority of peptide variants from protein N were found for non‐human‐viruses, variability was found also among human‐virus sequences: peptide AYNVTQAFGR has one variant, peptide GPEQTQGNFGDQELIR has two variants, peptide WYFYYLGTGPEAGANK has three variants, and peptides NPANNAAIVLQLPQGTTLPK, IGMEVTPSGTWLTYTGAIK, ADETQALPQR have four variants. Peptide GFYAEGSR was the only peptide from protein N which did not present any sequence variant. Thus, seven peptides could be interesting ultra‐conserved candidates. Among the five peptides from the 7–25 min window, four peptides are well‐conserved and only one possible variant would have to be monitored if necessary (one HTPINLVR variant from a non‐human virus).

**Figure 2:**
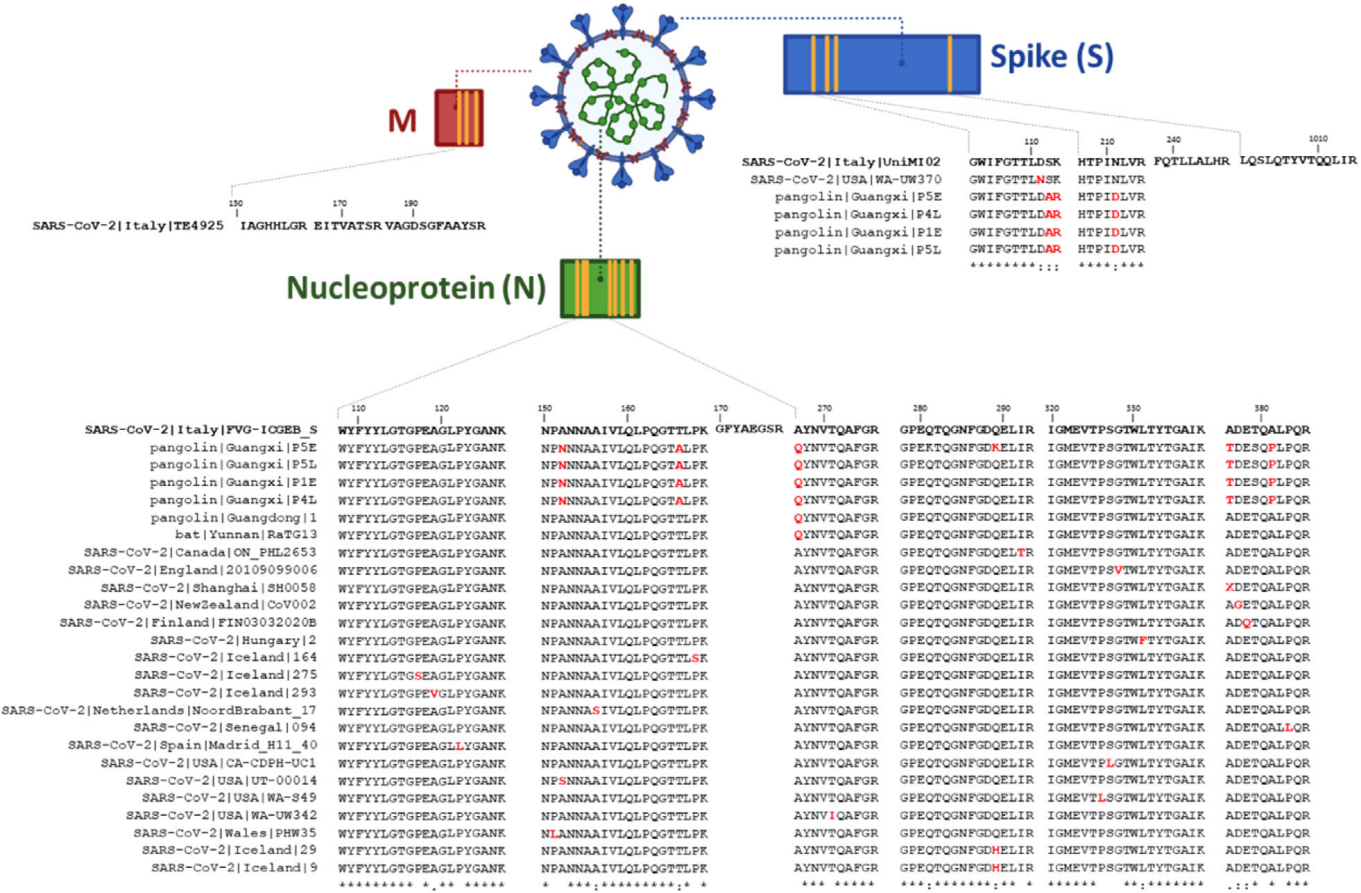
Sequence variants identified for each of the 14 peptides from Table 1. The sequences of these peptides are indicated in bold. Variants were identified through multiple sequence alignments of the ten viral proteins from virus Italy‐INMI1 strain with other sequenced human SARS‐CoV‐2, bat, and pangolin closely related viruses. Variations in the amino acid sequences are highlighted in red.

Table 1 also shows some additional parameters for guiding further the selection of peptides for targeted assays. Notably, we provide the potential susceptibility of each peptide to be modified, by looking into our DDA data, and through searching the peptide sequences in the Global Proteome Machine Database (GPMDB, https://www.thegpm.org/index.html) and checking the number of occurrences of modifications in other datasets containing these peptides. As detailed in the table, four peptides are more prone to modifications, peptides IGMEVTPSGTWLTYTGAIK, WYFYYLGTGPEAGLPYGANK, and NPANNAAIVLQLPQGTTLPK from protein N, and peptide GWIFGTTLDSK from protein S, respectively. In terms of occurrence of missed cleavages in the DDA data, six peptide sequences are present as one single sequence: IAGHHLGR, FQTLLALHR, GWIFGTTLDSK, HTPINLVR, LQSLQTYVTQQLIR, and IGMEVTPSGTWLTYTGAIK. Peptide ADETQALPQR appears to be the most susceptible to the occurrence of missed cleavages, with four different sequences observed in the data: ADETQALPQRQK, ADETQALPQRQKK, KADETQALPQRQR, KADETQALPQRQRQK, KKADETQALPQR. Furthermore, as observed in Figure 2, these peptides are belonging to potential mutation hotspot zones being also more prone to residue changes.

In summary, this dataset provides new data on SARS‐CoV‐2 protein and peptide sequences that could be of use for future developments of targeted assays for the detection of the virus in medical samples. Based on our experimental data and decision criteria, we consider that the best theoretical candidates for this purpose would be peptides LQSLQTYVTQQLIR, FQTLLALHR, HTPINLVR from protein S, along with peptide VAGDSGFAAYSR from protein M. Nevertheless, peptides ADETQALPQR, AYNVTQAFGR, NPANNAAIVLQLPQGTTLPK, and WYFYYLGTGPEAGLPYGANK from protein N, due to their specificity for SARS‐CoV‐2, could be of use if one takes into account their variants and their structural modifications. Peptides EITVATSR and IAGHHLGR (protein M) and peptide GFYAEGSR (protein N) showed good analytical characteristics but unfortunately their sequences are shared with other eukaryotes and bacteria. They can still be used for targeted method development but only in the presence of another peptide from the same protein which is specific to SARS‐CoV‐2. Taking into account the good signals obtained for these peptides in the presence of a background matrix in DDA mode, we expect that some peptides can be detected in real clinical samples by using sensitive targeted approaches, but we recommend specific rigorous studies to establish the limit of detection of any methodology. We believe this study reinforces and complements some currently on‐going projects that aim at providing MS‐based tools for use in diagnostic of SARS‐CoV‐2, but until now unpublished.^[^ ^13^, ^14^, ^15^ ^]^ To our knowledge, only one proteomic dataset related to COVID‐19 has been published,^[^ ^16^ ^]^ presenting an interactomics study with viral proteins individually produced in host cells. The present dataset is the first published work on proteomics carried out with SARS‐CoV‐2 virus. Compared to other on‐going studies, the information provided here covers important analytical characteristics of peptides from the different structural viral proteins for the development of MS‐based diagnostic. We present the conservation of peptides among the different types of SARS‐CoV‐2 sequenced up until recently because mutations naturally occurring in the viral genome upon its spread may challenge molecular biology diagnostic tests. Furthermore, the search of naturally attenuated variants that has been recently proposed as an interesting perspective^[^ ^17^ ^]^ could be carried out with a systematic search of these peptides. This experimental dataset allows researchers to use this information in order to select and develop the most appropriate MS methods for the detection of the virus. All the MS raw files and peptide search results are made available for researchers to mine the data and search for other relevant peptides that could be of use for their studies.

## Conflict of Interest

The authors declare no conflict of interest.

## Author Contributions

D.G., L.G., O.P., and J.A. conceived the study. J.C.G. performed the proteomic experimental work. F.G. and L.B. contributed the biological material. D.G., L.G., J.C.G., O.P., and J.A. analyzed the data. D.G., L.G., and J.A. wrote the manuscript with help from all the co‐authors.

## Supporting information

Supporting Table S1

Supporting Table S2

Supporting Table S3

Supporting Information
