# Supplementary material for: Shortlisting SARS‐CoV‐2 Peptides for Targeted Studies from Experimental Data‐Dependent Acquisition Tandem Mass Spectrometry Data
Source: Proteomics. 2020 Jun 21;20(14):2000107. doi: 10.1002/pmic.202000107 (PMC7267140; doi:10.1002/pmic.202000107)
Supplement: Supplementary file 4 — Supporting Information [file PMIC-20-0-s002.pptx]

## Slide 1
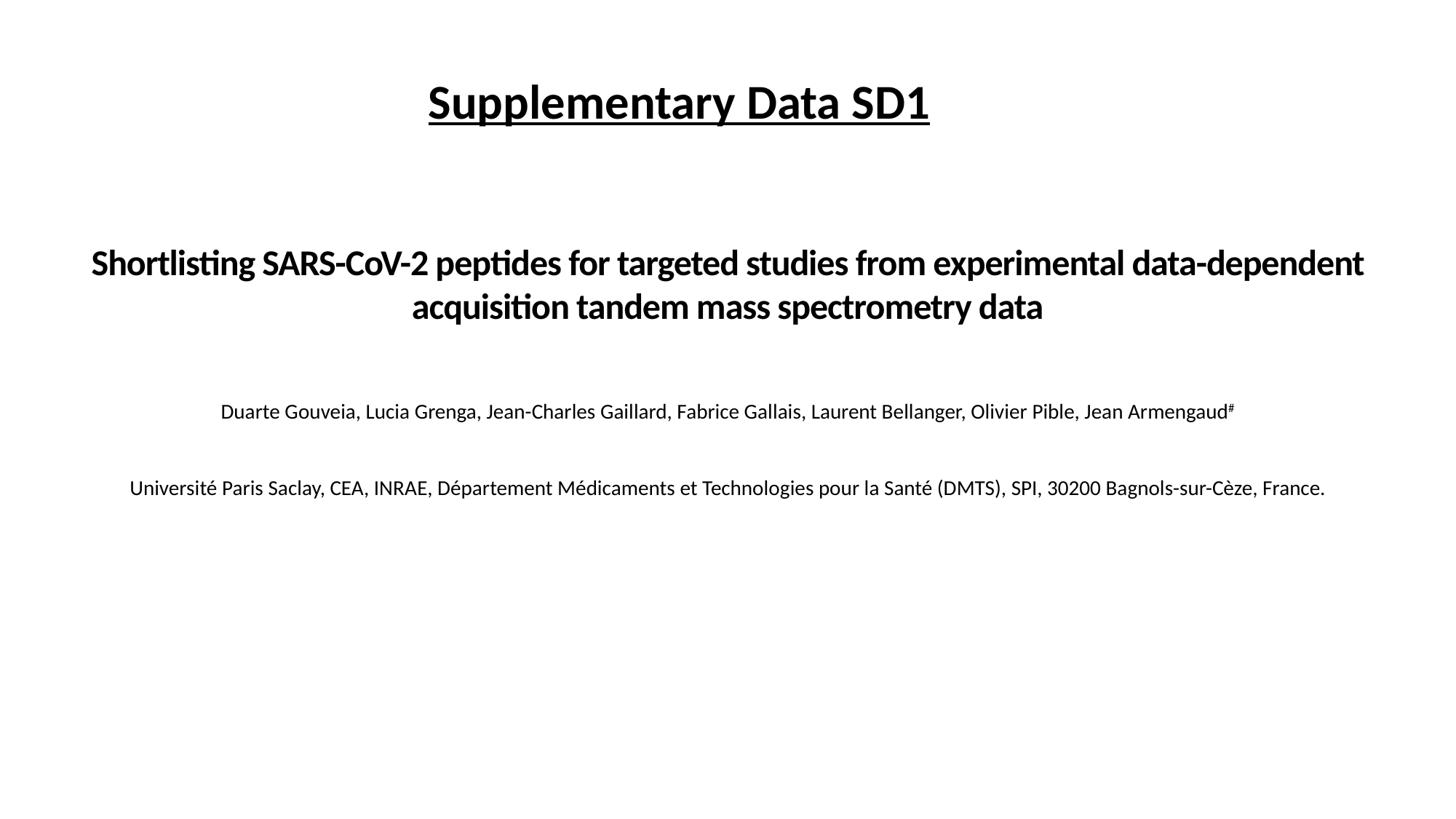

Supplementary Data SD1
Shortlisting SARS-CoV-2 peptides for targeted studies from experimental data-dependent acquisition tandem mass spectrometry data
Duarte Gouveia, Lucia Grenga, Jean-Charles Gaillard, Fabrice Gallais, Laurent Bellanger, Olivier Pible, Jean Armengaud#
Université Paris Saclay, CEA, INRAE, Département Médicaments et Technologies pour la Santé (DMTS), SPI, 30200 Bagnols-sur-Cèze, France.

## Slide 2
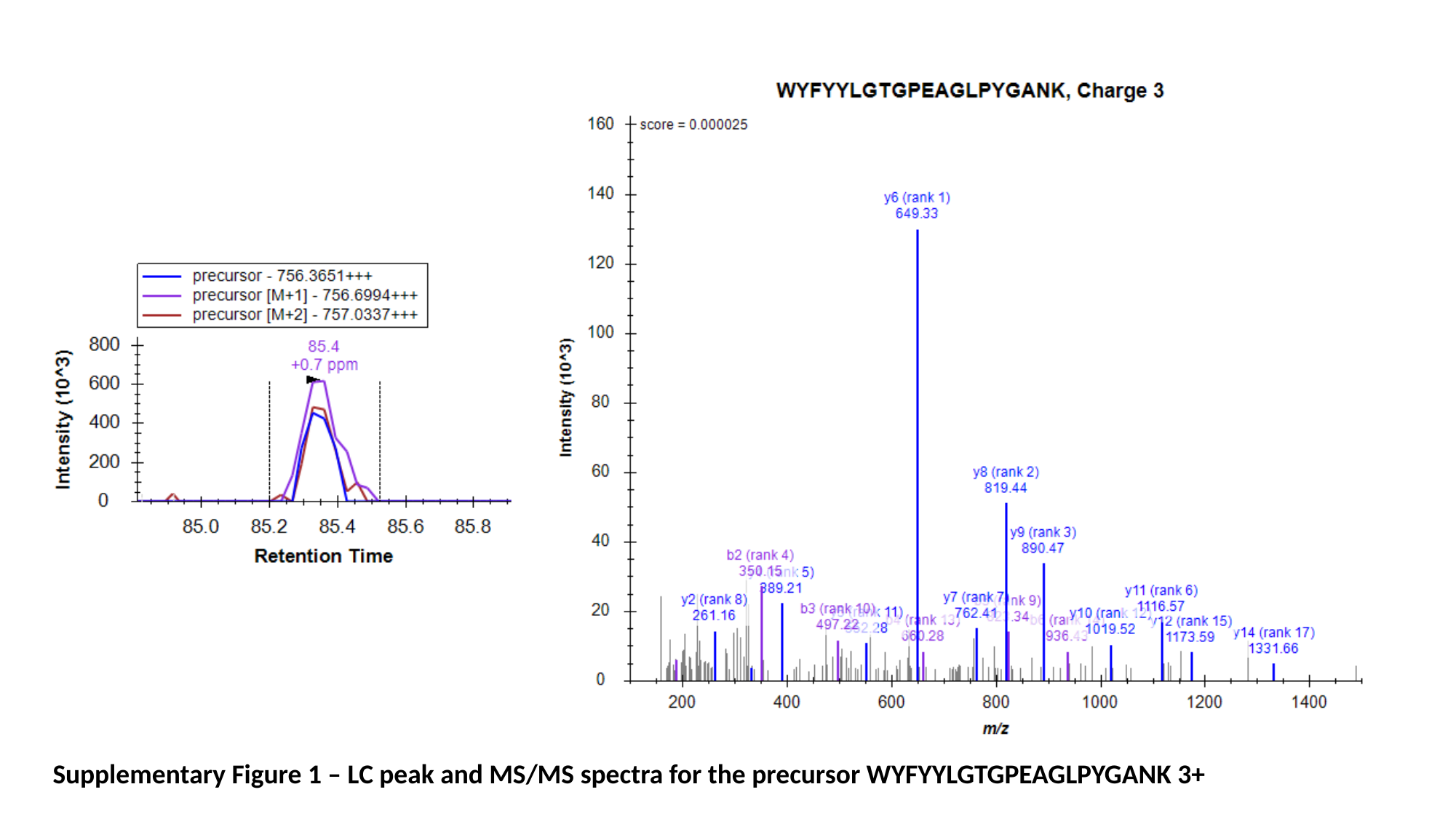

Supplementary Figure 1 – LC peak and MS/MS spectra for the precursor WYFYYLGTGPEAGLPYGANK 3+

## Slide 3
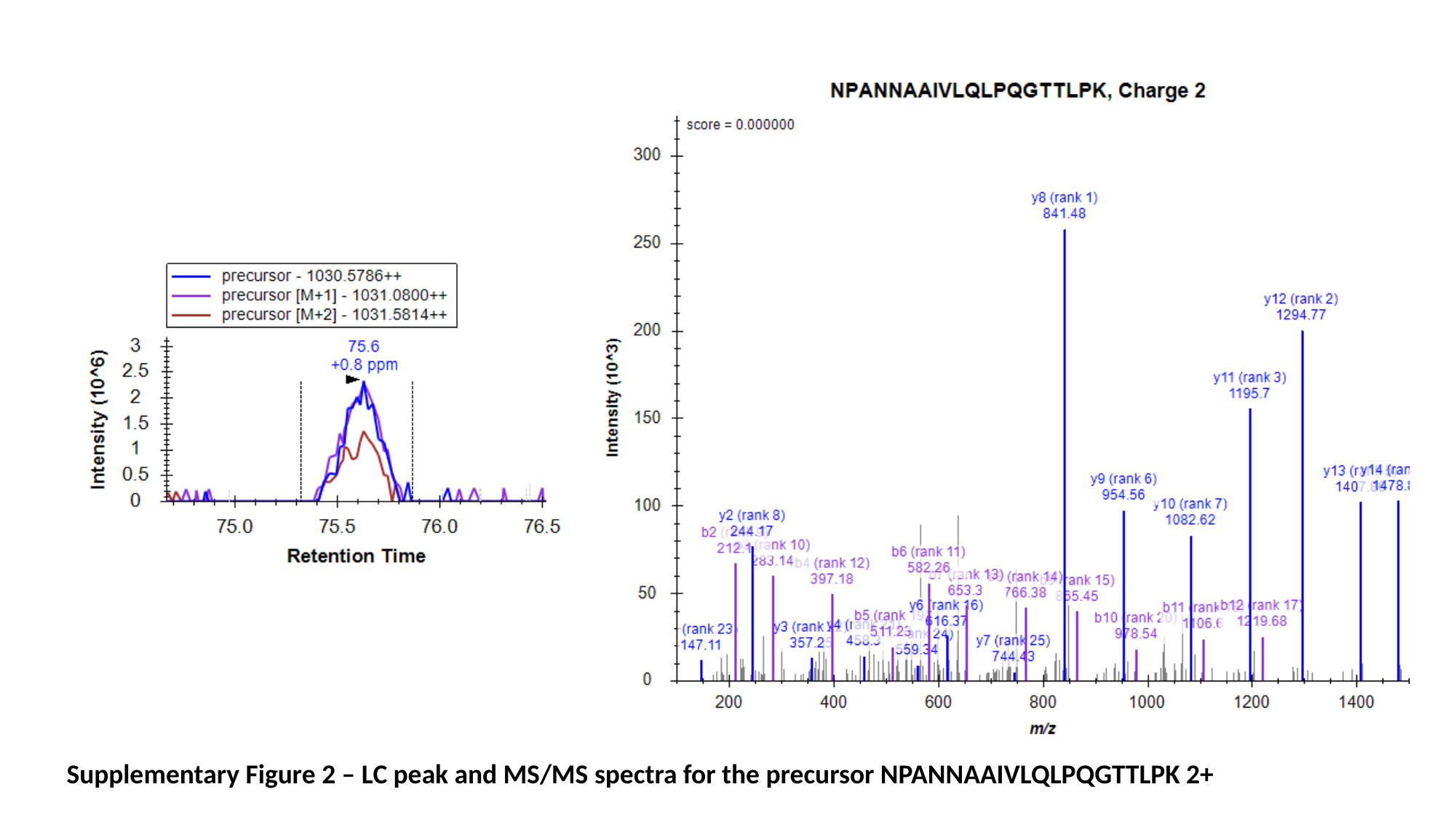

Supplementary Figure 2 – LC peak and MS/MS spectra for the precursor NPANNAAIVLQLPQGTTLPK 2+

## Slide 4
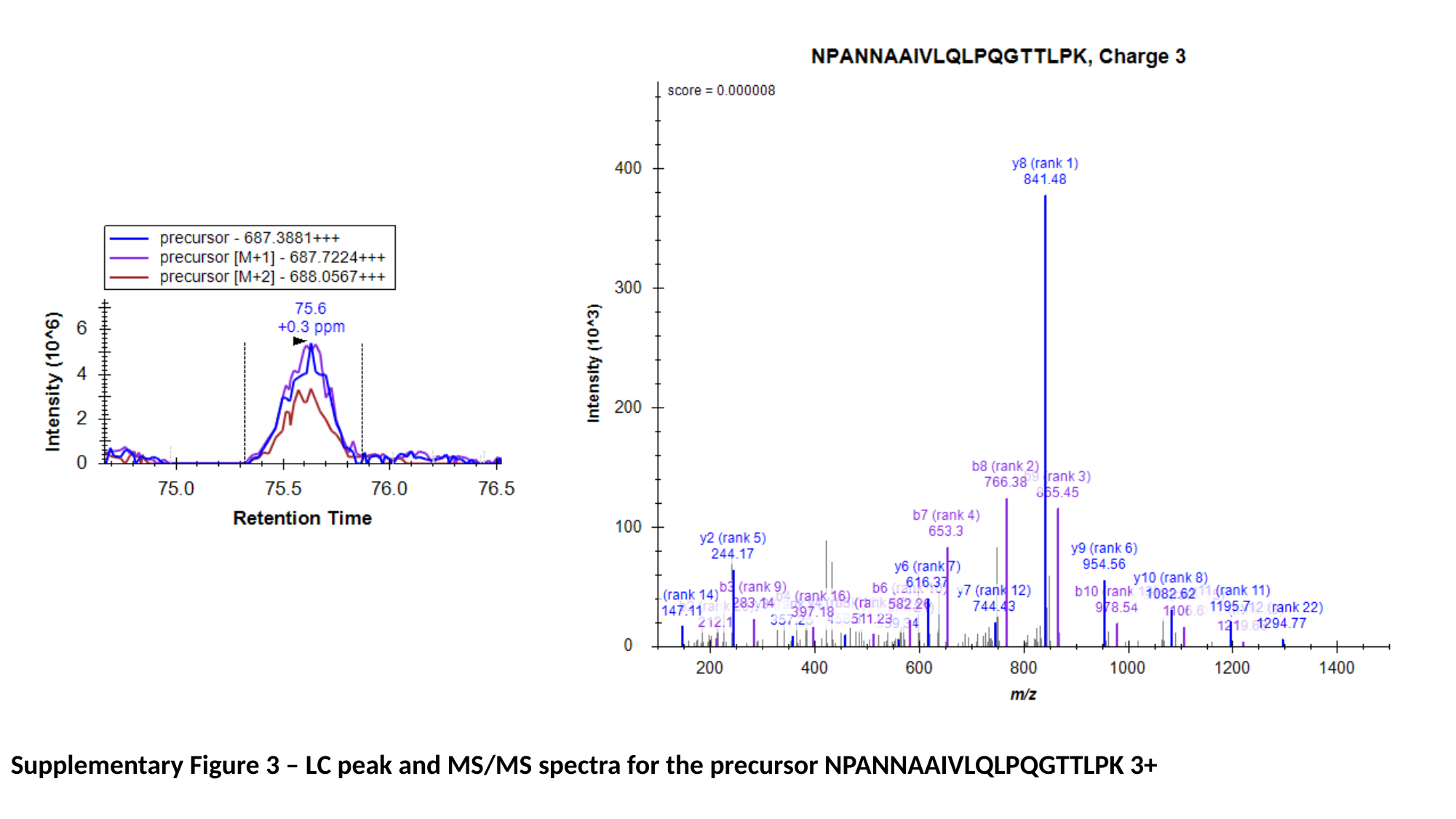

Supplementary Figure 3 – LC peak and MS/MS spectra for the precursor NPANNAAIVLQLPQGTTLPK 3+

## Slide 5
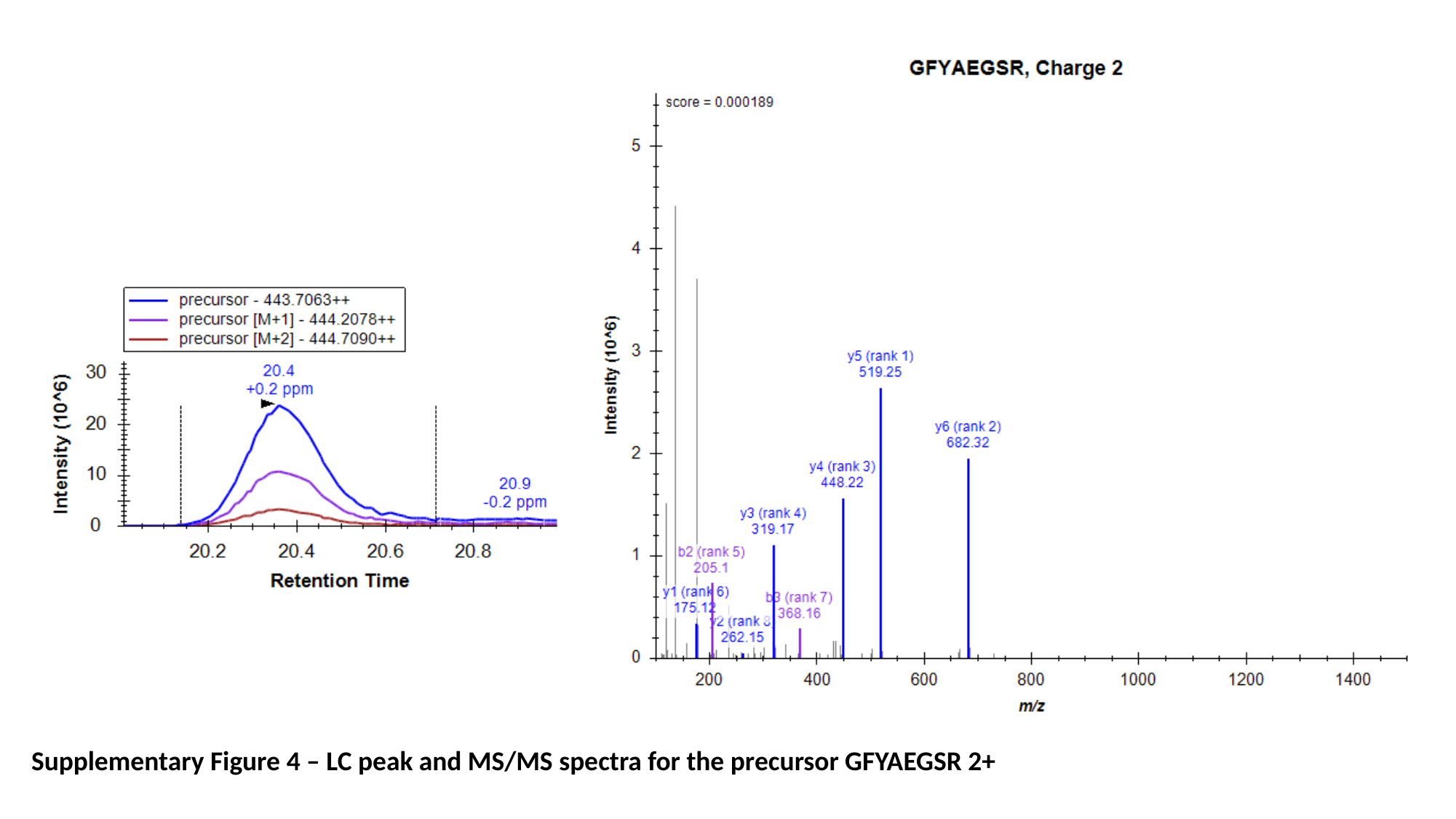

Supplementary Figure 4 – LC peak and MS/MS spectra for the precursor GFYAEGSR 2+

## Slide 6
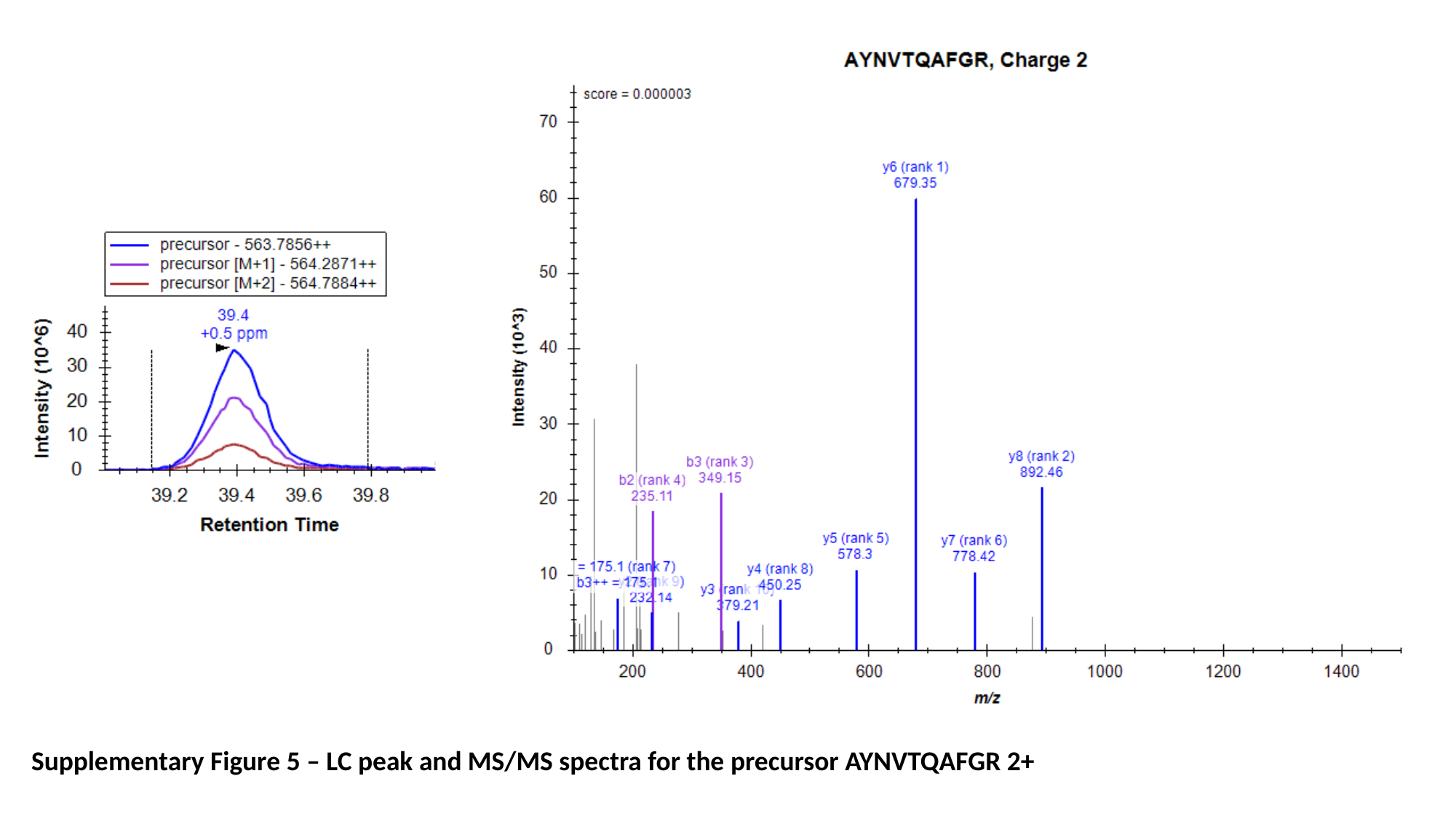

Supplementary Figure 5 – LC peak and MS/MS spectra for the precursor AYNVTQAFGR 2+

## Slide 7
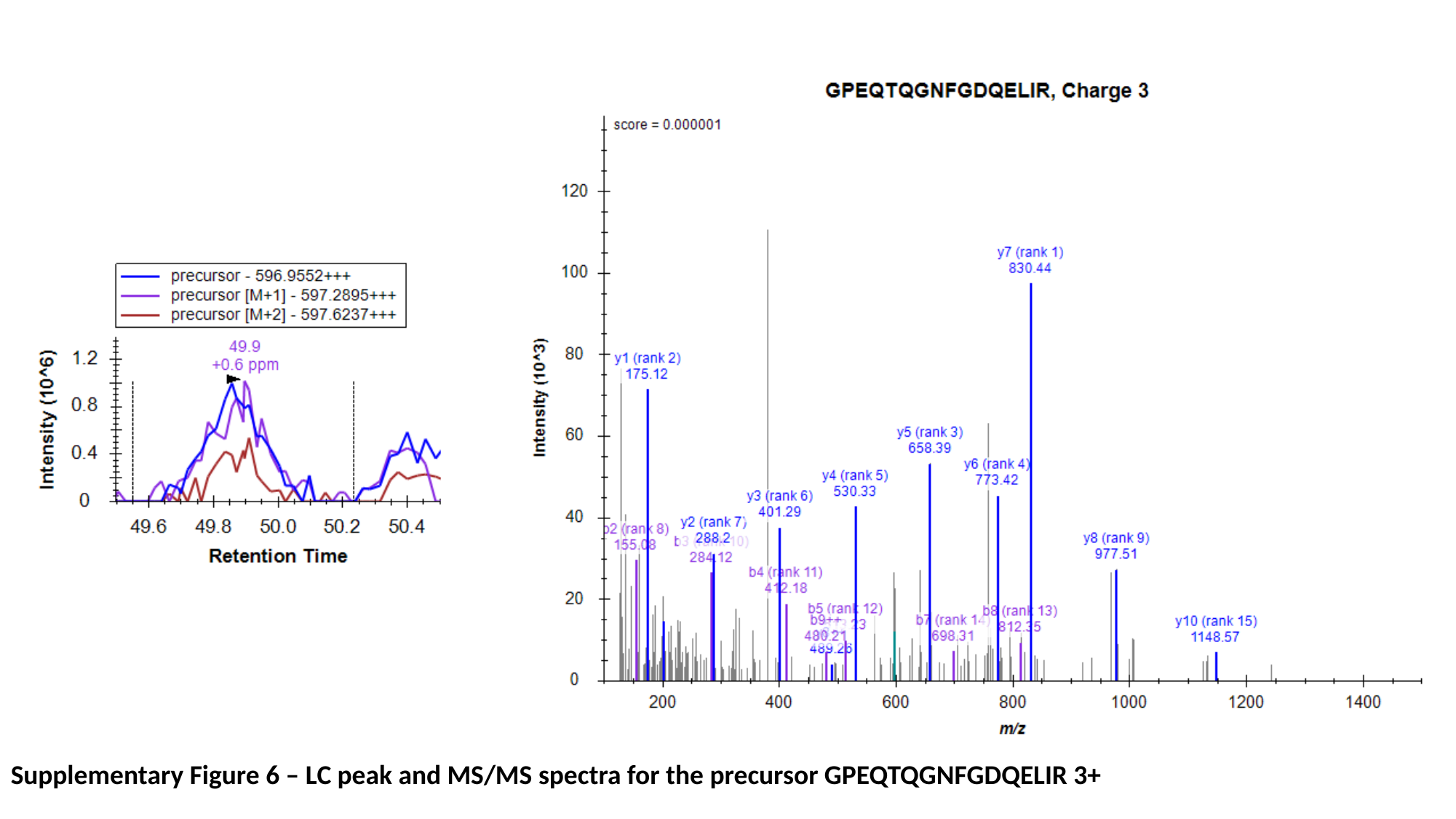

Supplementary Figure 6 – LC peak and MS/MS spectra for the precursor GPEQTQGNFGDQELIR 3+

## Slide 8
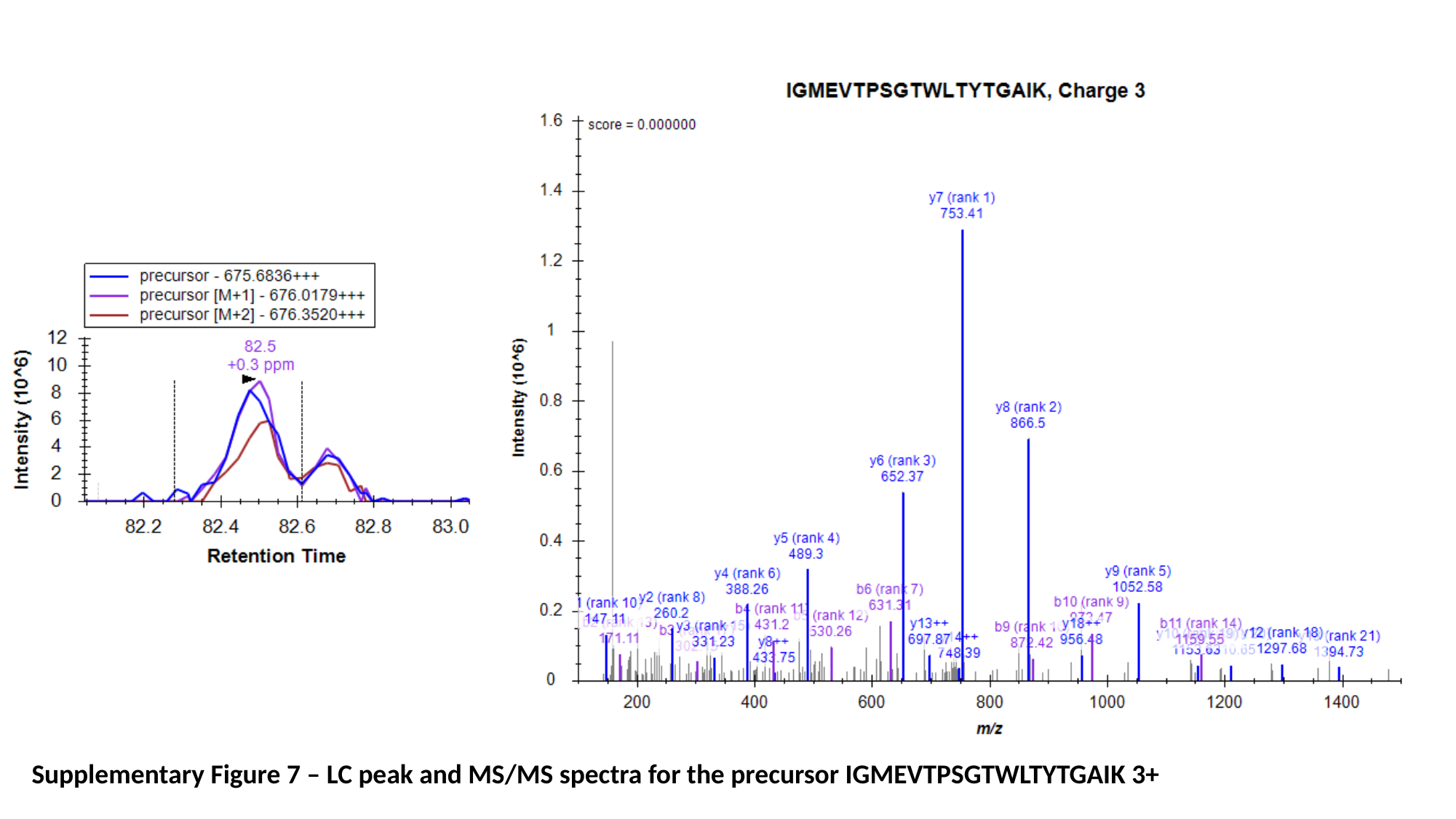

Supplementary Figure 7 – LC peak and MS/MS spectra for the precursor IGMEVTPSGTWLTYTGAIK 3+

## Slide 9
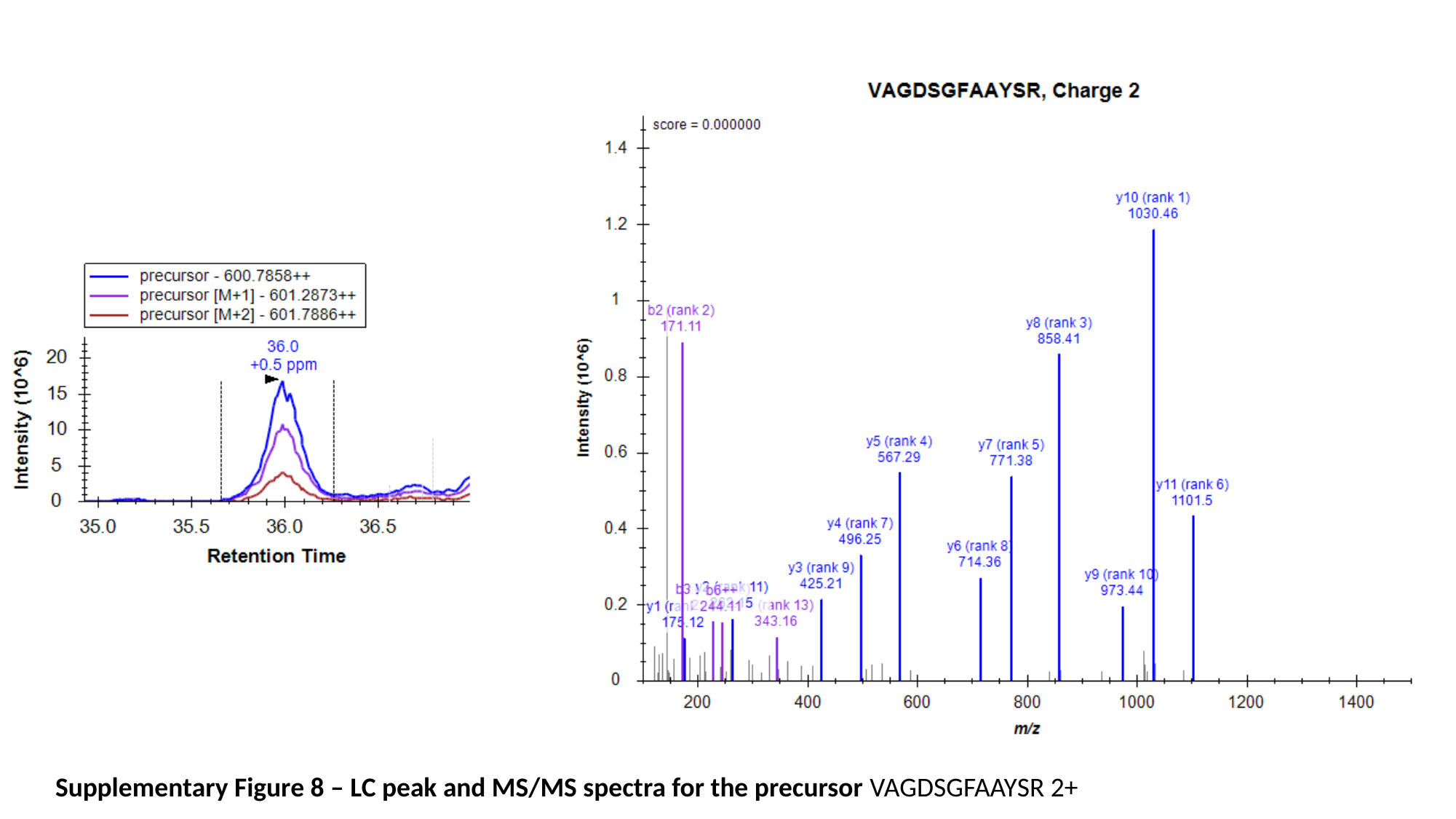

Supplementary Figure 8 – LC peak and MS/MS spectra for the precursor VAGDSGFAAYSR 2+

## Slide 10
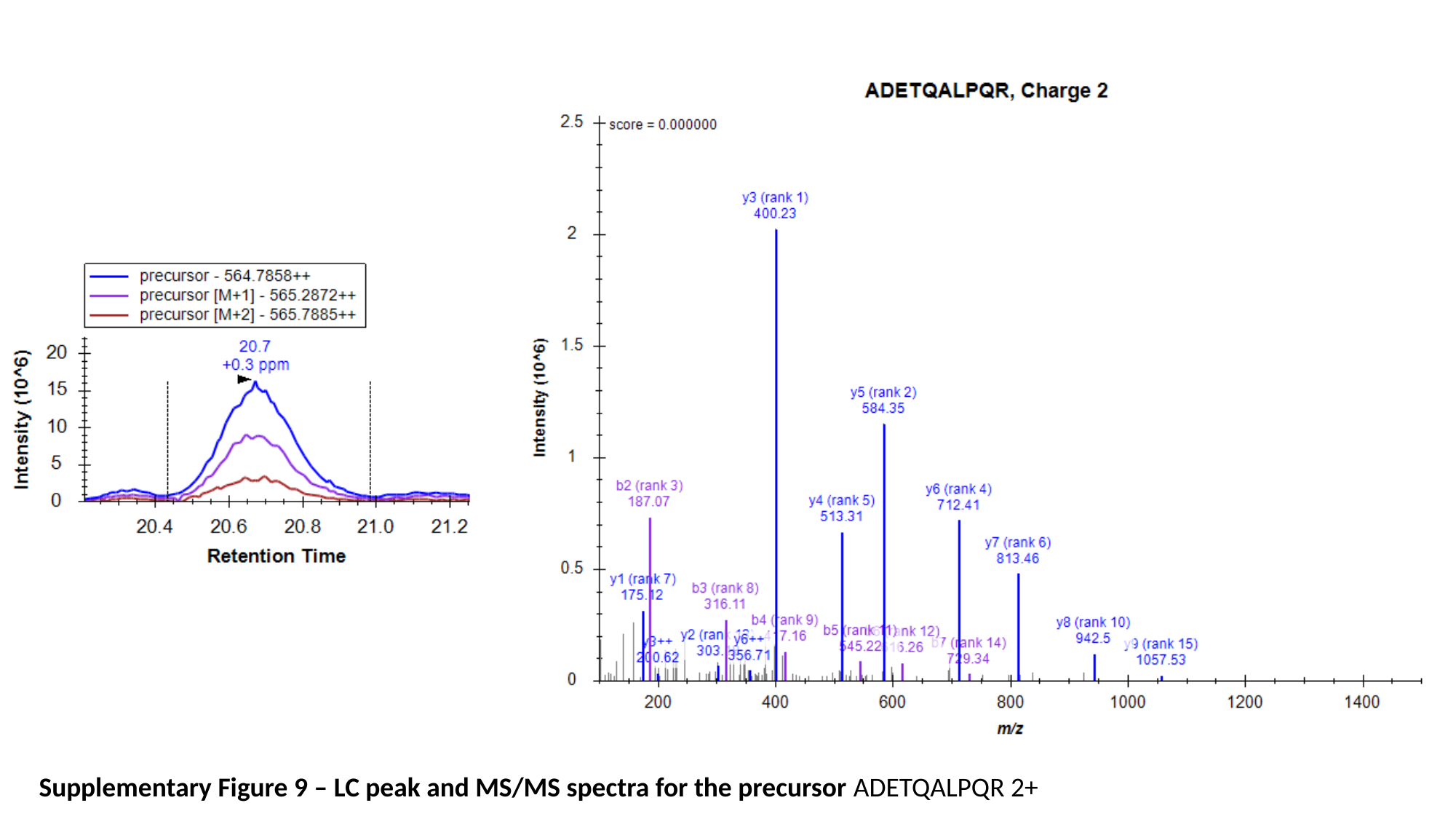

Supplementary Figure 9 – LC peak and MS/MS spectra for the precursor ADETQALPQR 2+

## Slide 11
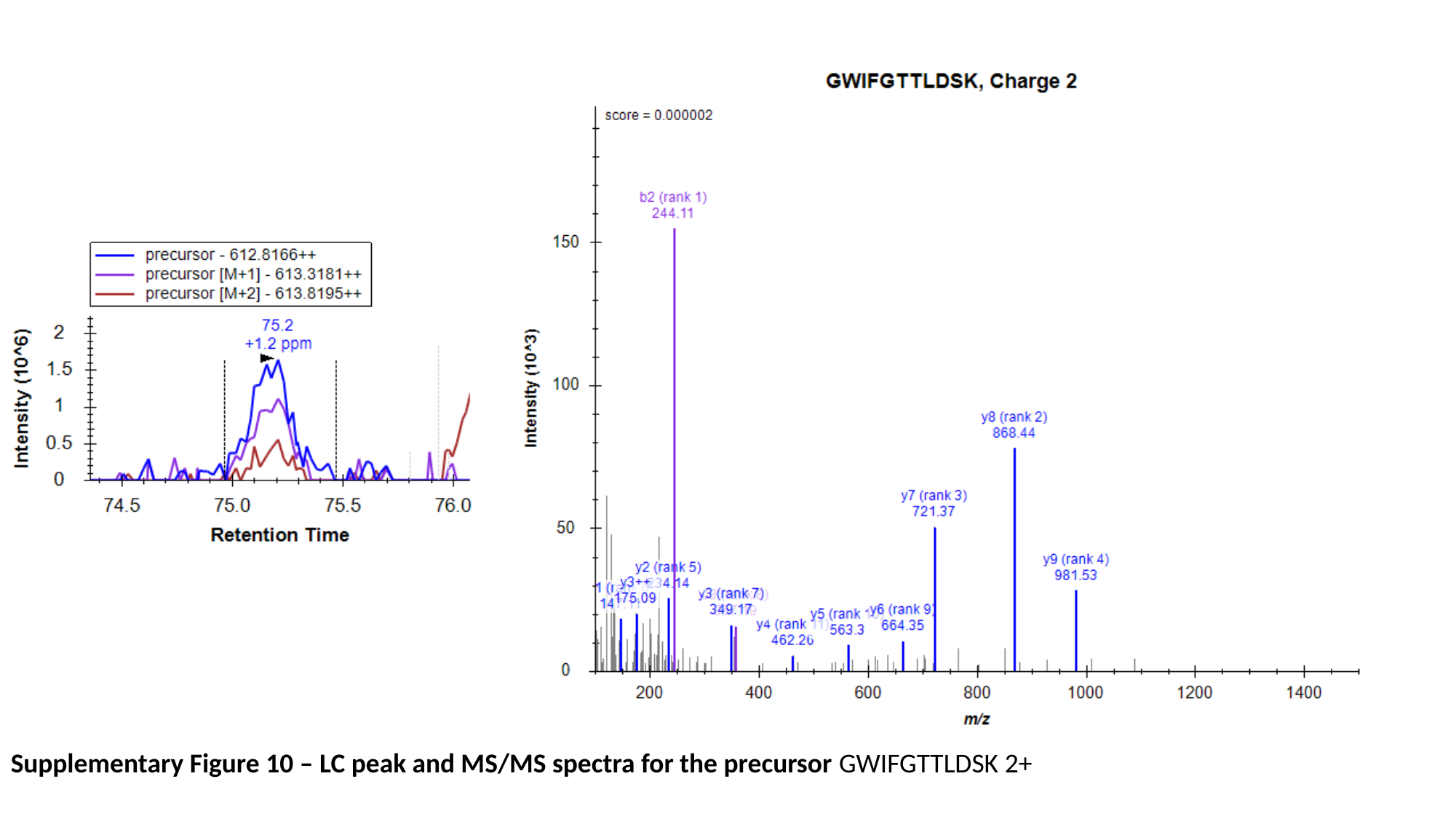

Supplementary Figure 10 – LC peak and MS/MS spectra for the precursor GWIFGTTLDSK 2+

## Slide 12
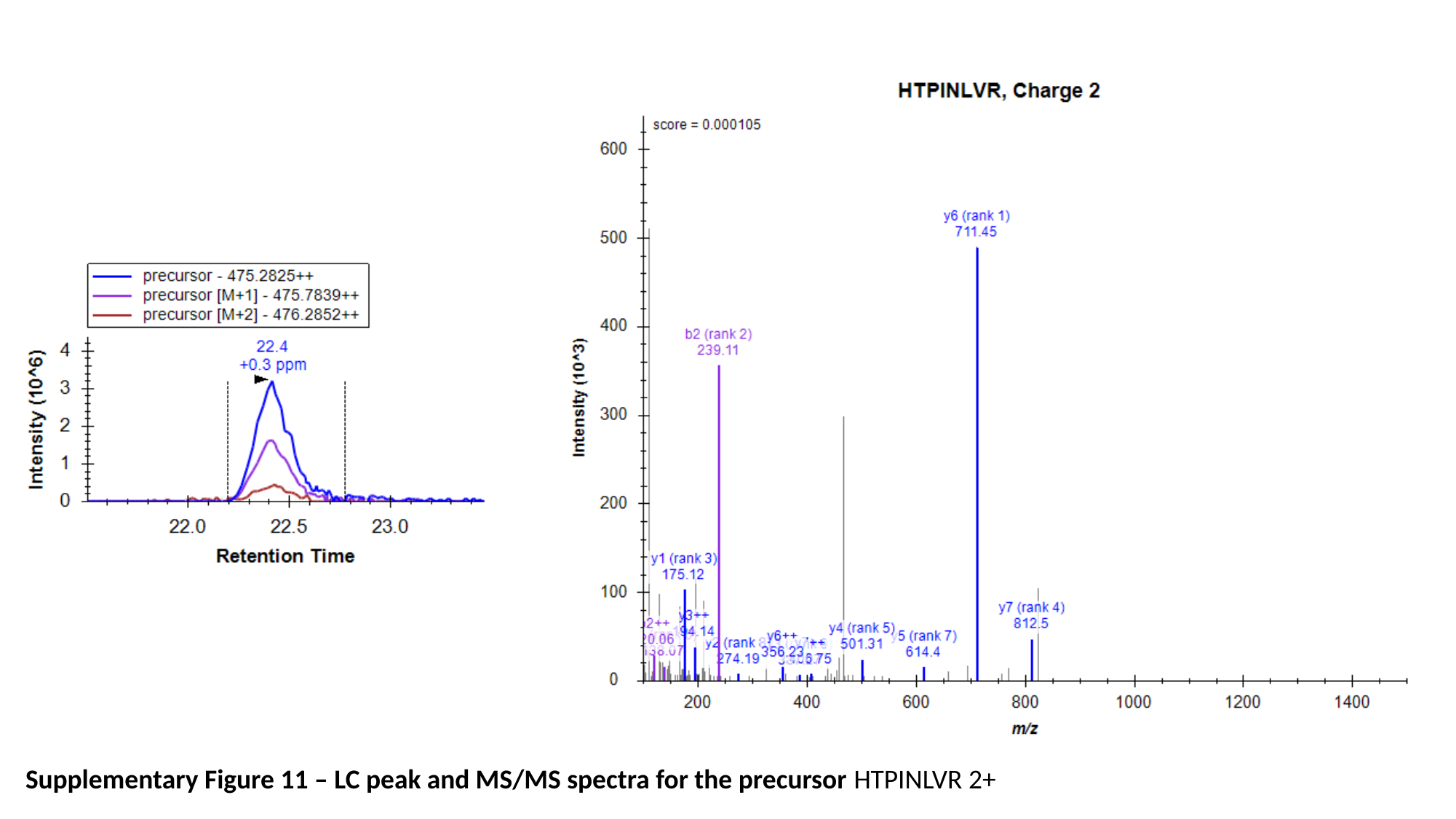

Supplementary Figure 11 – LC peak and MS/MS spectra for the precursor HTPINLVR 2+

## Slide 13
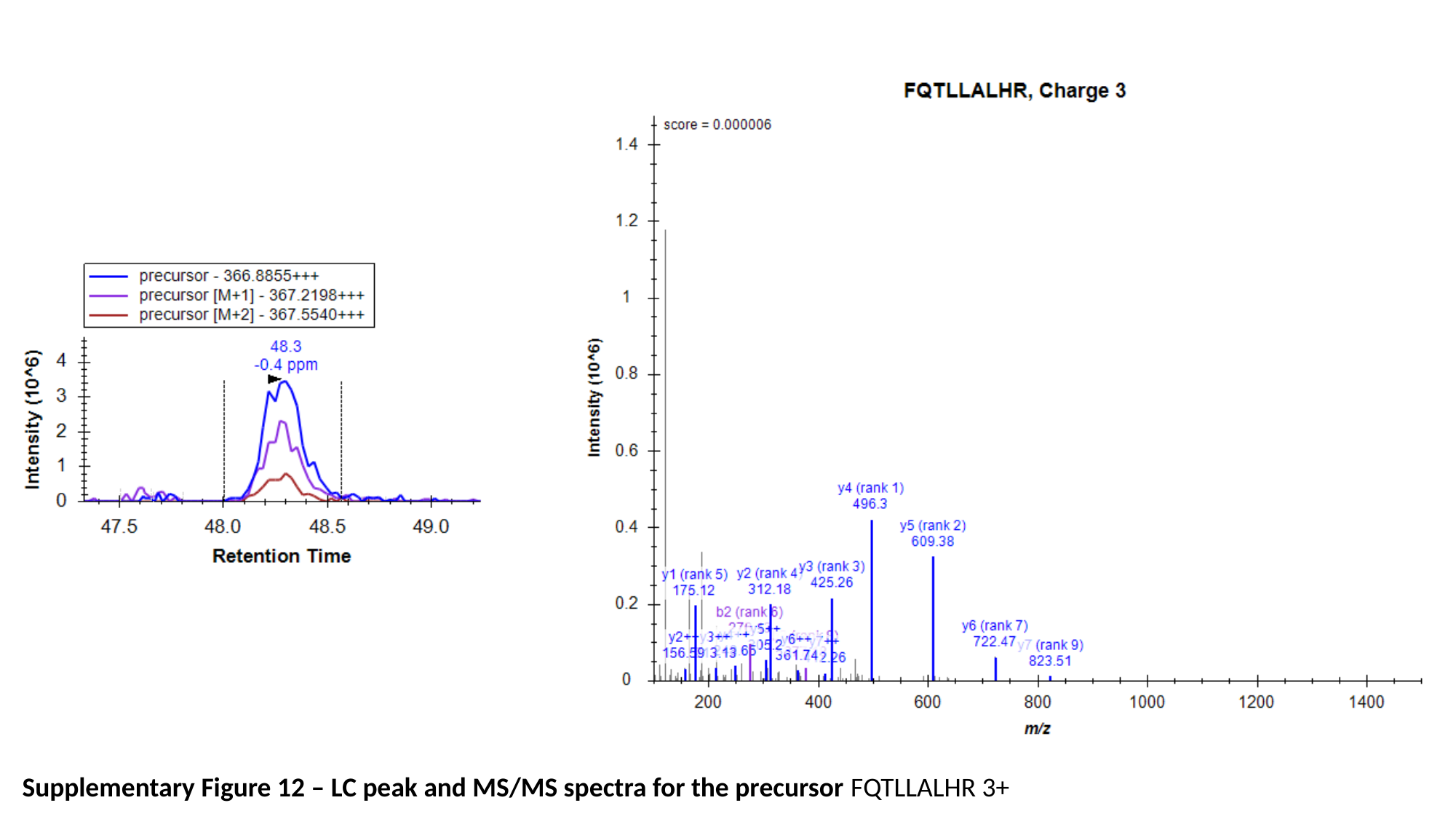

Supplementary Figure 12 – LC peak and MS/MS spectra for the precursor FQTLLALHR 3+

## Slide 14
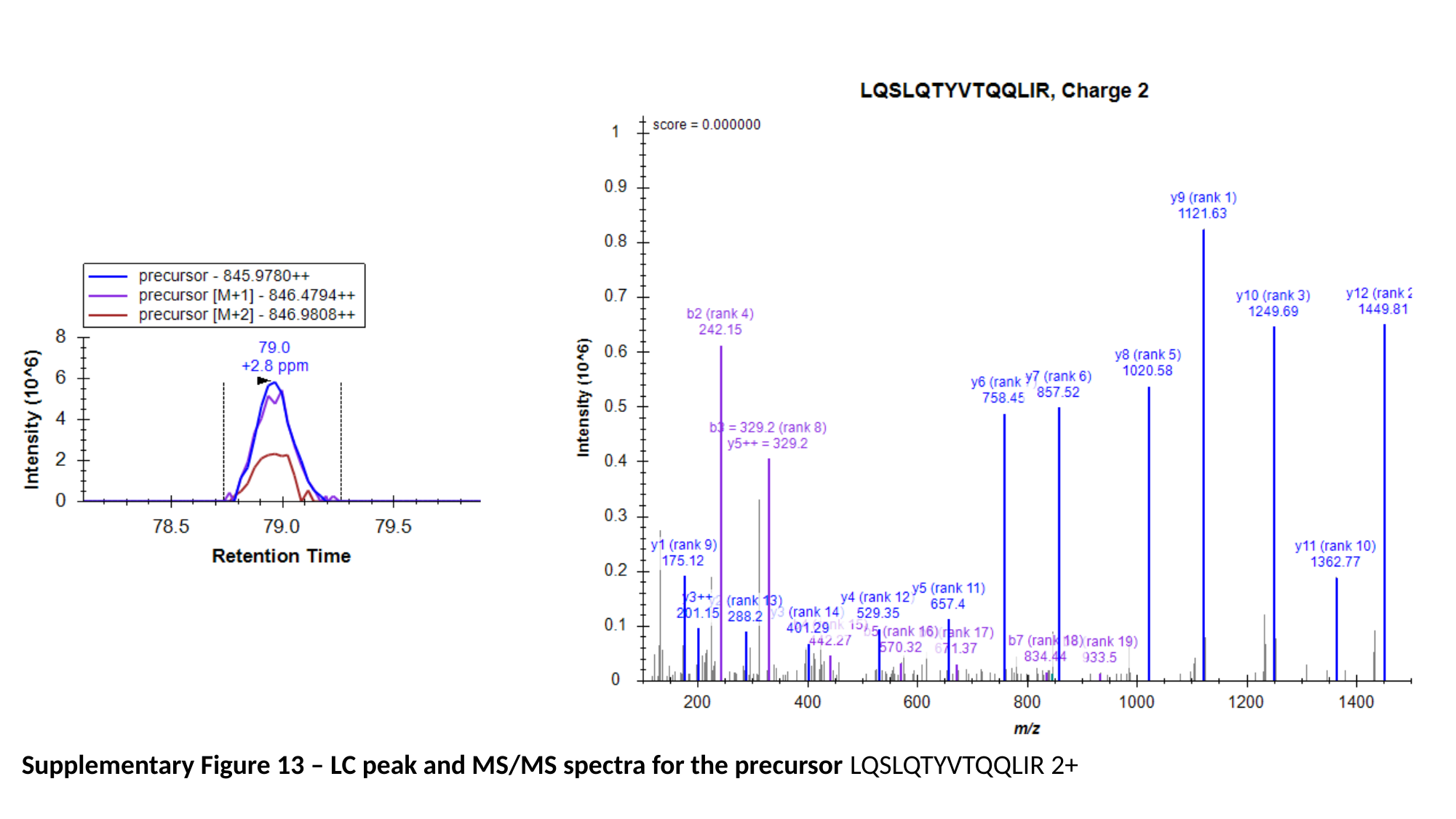

Supplementary Figure 13 – LC peak and MS/MS spectra for the precursor LQSLQTYVTQQLIR 2+

## Slide 15
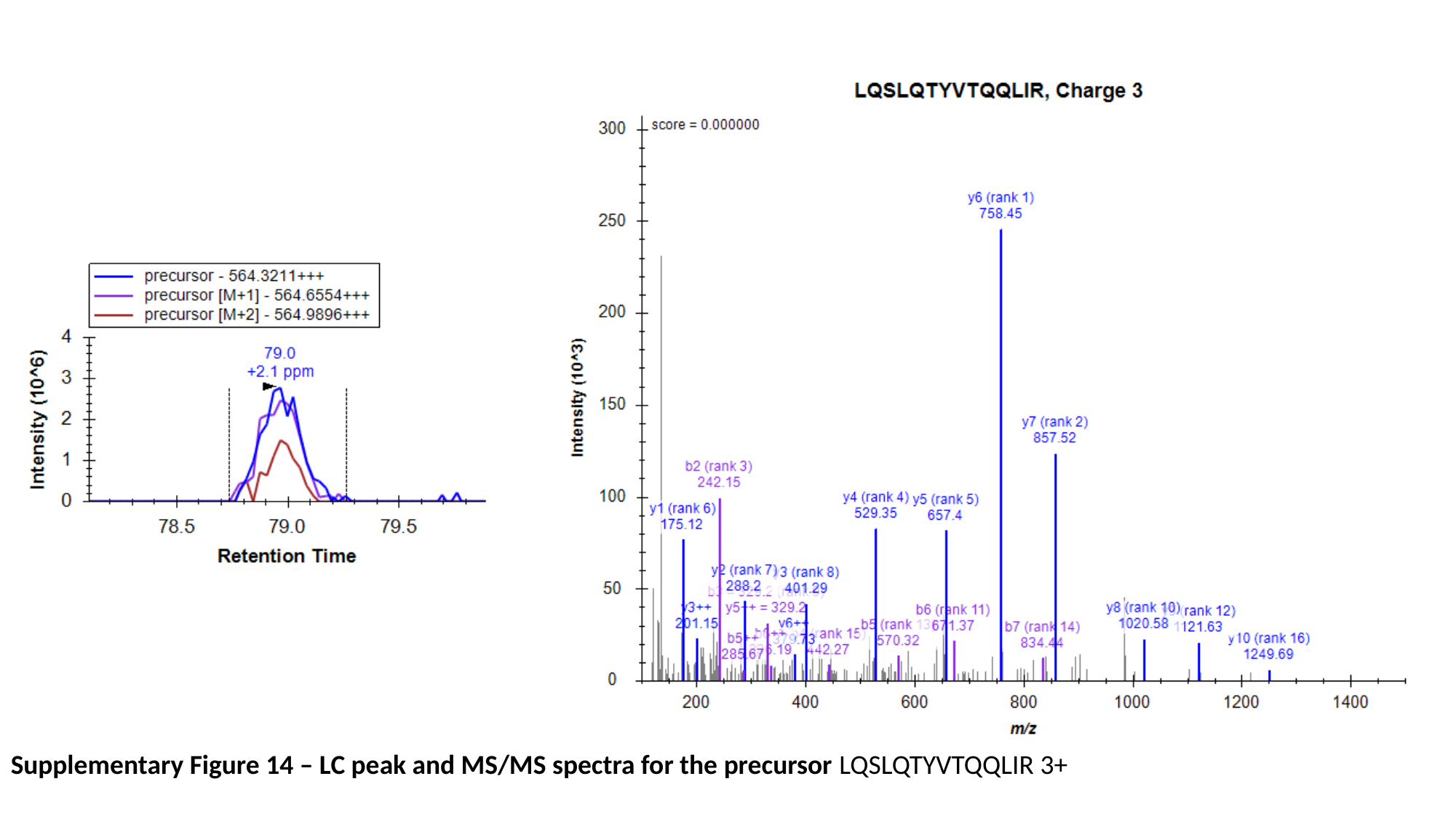

Supplementary Figure 14 – LC peak and MS/MS spectra for the precursor LQSLQTYVTQQLIR 3+

## Slide 16
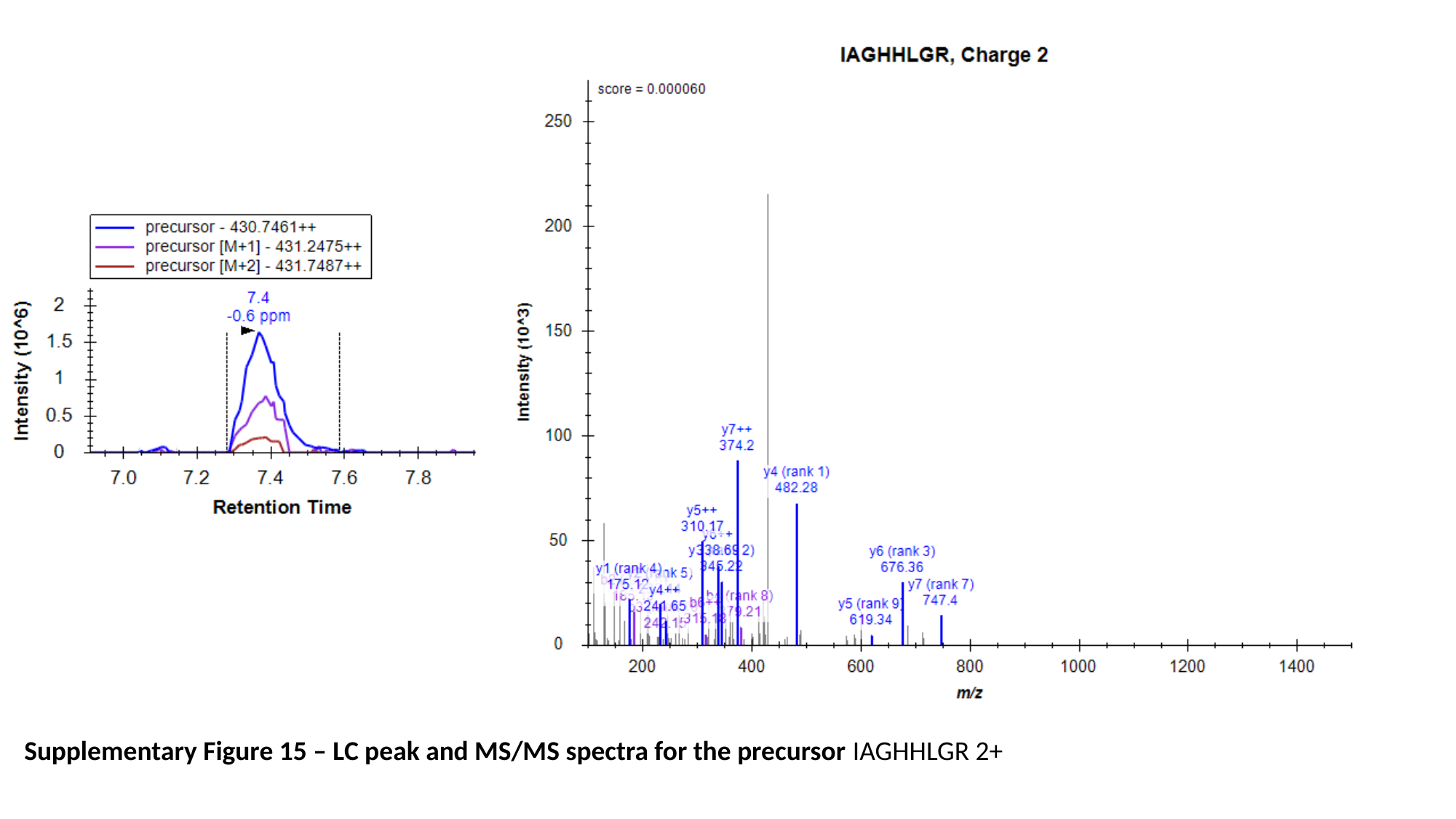

Supplementary Figure 15 – LC peak and MS/MS spectra for the precursor IAGHHLGR 2+

## Slide 17
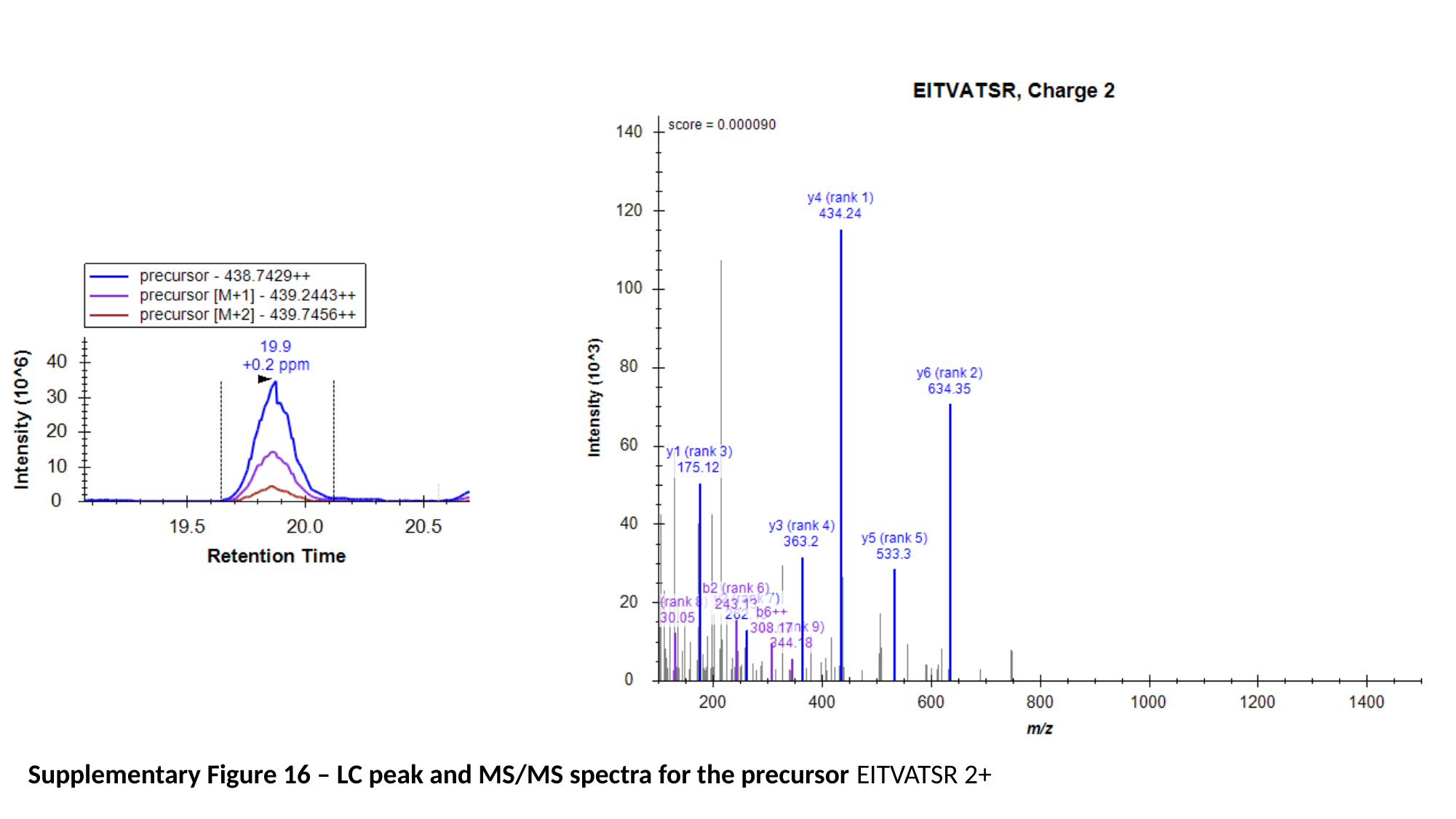

Supplementary Figure 16 – LC peak and MS/MS spectra for the precursor EITVATSR 2+
